# Introducing Digital Pharmacometrics: A Pharmacokinetics (PK)/Pharmacodynamics (PD) Framework for Prescription Digital Therapeutics

**DOI:** 10.7759/cureus.84195

**Published:** 2025-05-15

**Authors:** Shaheen E Lakhan

**Affiliations:** 1 Medical Office, Click Therapeutics, Inc., New York, USA; 2 Department of Neurology, Western University of Health Sciences, Pomona, USA; 3 Department of Neurology, A.T. Still University School of Osteopathic Medicine in Arizona, Mesa, USA; 4 Department of Neurology, Morehouse School of Medicine, Atlanta, USA; 5 Department of Bioscience, Global Neuroscience Initiative Foundation, Miami, USA

**Keywords:** adme framework, behavioral interventions, closed-loop therapeutics, digital dose, digital exposure, digital pharmacodynamics, digital pharmacokinetics, digital working alliance, prescription digital therapeutics, software-based medicine

## Abstract

Prescription digital therapeutics (PDTs) are FDA-authorized, clinically validated software applications that deliver behavioral and cognitive interventions through digital platforms. While these therapeutics are prescribed and regulated like drugs, they lack an established pharmacologic framework to define how digital content is delivered, processed, and translated into clinical benefit. In this editorial, we draw on rich experience in drug and PDT development to introduce the emerging discipline of digital pharmacometrics: the application of pharmacokinetics (PK) and pharmacodynamics (PD) principles to software-based medicine. We redefine classical concepts such as dose, exposure, and the absorption, distribution, metabolism, and excretion (ADME) framework for the context of digital therapeutics and identify Digital Working Alliance (DWA) formation as a critical modulator of therapeutic bioavailability. We also characterize digital PD through the development of dose-exposure-response curves that capture behavioral, biometric, and clinical outcomes, enabling closed-loop, adaptive therapeutic systems. This digital PK/PD model supports better trial design, labeling clarity, regulatory oversight, clinical guidance, and reimbursement models. By formally codifying how PDTs interact with patients and produce effects, digital pharmacometrics provides the scientific foundation necessary to scale precision software medicine.

## Editorial

From molecules to modules

Pharmacokinetics (PK) and pharmacodynamics (PD) are the conceptual backbone of modern medicine. PK describes how a drug is absorbed, distributed, metabolized, and excreted by the body, while PD describes what the drug does to the body in terms of mechanism of action, efficacy, and safety. These models underpin nearly every decision in drug development and clinical pharmacology: dosage forms, labeling language, prescriber guidance, and reimbursement strategy.

Yet as software-based medicine matures, a new therapeutic modality is challenging this molecular paradigm. Prescription digital therapeutics (PDTs) are FDA-authorized, clinically validated software applications that deliver structured, interactive interventions through smartphones [1]. Unlike wellness apps or coaching platforms, PDTs are regulated as medical devices and often evaluated through randomized controlled trials. They target conditions ranging from major depressive disorder to migraine, often complementing traditional pharmacological therapy [1].

Despite this progress, PDTs remain structurally disadvantaged by the absence of an accepted PK/PD analog. In the pharmacological world, terms like bioavailability, half-life, and dose-response have well-understood operational definitions. No such language yet exists to describe how a digital therapeutic moves through and affects the human system. This lack of structure impedes regulatory clarity, hinders trial design, undermines labeling consistency, and slows payer adoption. Without a pharmacometric framework, software-based therapeutics risk being evaluated with language designed for chemicals.

In this editorial, we apply our deep expertise in both drug and PDT development of several first-in-class and first-in-disease therapies to introduce the concept of digital pharmacometrics, a discipline that reinterprets PK/PD principles for the software era. We begin by defining digital PK in terms of dose, exposure, and an adapted absorption, distribution, metabolism, and excretion (ADME) framework. We then characterize digital PD through software-mediated effects on brain, behavior, and function, culminating in closed-loop therapeutic systems. Finally, we examine implications across clinical development, regulatory strategy, commercial models, and real-world clinical practice. Our aim is to initiate a field of digital pharmacometrics, bringing scientific rigor and shared language to a transformative class of therapeutics.

Digital pharmacokinetics: digital dose, exposure, and ADME

The PK of a drug maps how it moves through the body over time [2]. For PDTs, the challenge is mapping how structured therapeutic software moves through the mind and behavior of the patient. We begin with two foundational concepts: digital dose and digital exposure.

Digital dose refers to the prescribed interaction with the PDT. This includes session frequency, duration, module order, and adaptive pathways. For example, a PDT for migraine prevention may recommend daily 10-15-minute sessions per week for 12 weeks, delivered through a sequence of neuro-cognitive and -behavioral modules. Like drug regimens, digital doses can be fixed or personalized, titrated over time, and designed to minimize side effects like digital fatigue.

Digital exposure reflects what the patient actually experiences. While the dose is prescribed, exposure is realized. A patient may skip sessions, interact passively, or engage deeply. Exposure includes timing, environment, cognitive effort, affective resonance, and biometric signals such as heart rate variability. Understanding exposure is essential to explaining variability in clinical response and defining minimal effective use.

To structure digital PK, we retain the classical ADME scaffold [2] but redefine each term to match the software therapeutic context (Table 1). Absorption represents the extent to which the digital dose is initiated and behaviorally taken up. This includes app download, onboarding, and initial module engagement. Distribution captures the dispersion of therapeutic engagement across time, contexts, and internal states, encompassing time-of-day use, multitasking environments, and emotional or cognitive readiness. Metabolism reflects the transformation of content through personalization, symptom-based adaptation, or AI-driven tailoring. It shows how the software changes to fit the patient. Excretion refers to the decay of engagement, dropout, or cognitive washout, capturing both passive attrition and deliberate completion. This digital ADME framework provides a high-fidelity analog to classical PK, allowing structured evaluation of therapeutic delivery, retention, and engagement across populations.

**Table 1:** Digital Pharmacokinetics of Dose, Exposure, and ADME with Pharmacologic Comparison This author-created table presents a pharmacokinetically grounded framework for understanding how prescription digital therapeutics (PDTs) are prescribed, engaged with, and processed by patients. Classical pharmacology concepts of dose, exposure, absorption, distribution, metabolism, and excretion (ADME) are retained, but redefined to reflect software-based therapeutic mechanisms. A side-by-side comparison illustrates how each term translates from traditional drug delivery to neuro-cognitive and -behavioral engagement in PDTs.

Domain	Pharmacologic Definition	Digital Therapeutic Definition
Dose	The amount and schedule of a drug administered to achieve therapeutic levels.	The prescribed interaction pattern with the PDT, including module/session/task frequency, duration, sequence, and titration.
Exposure	The amount of drug that is actually absorbed and available at the site of action.	What the patient actually experiences, including timing, cognitive effort, emotional resonance, and context.
Absorption	The process by which a drug enters the bloodstream from its site of administration.	The initiation of the digital intervention, such as download, onboarding, and first module engagement.
Distribution	The dispersion or dissemination of substances throughout the fluids and tissues of the body.	The dispersion of interaction across time, settings, and patient states, such as time-of-day use, ecology, and multitasking environments.
Metabolism	The biotransformation of a substance, usually by liver enzymes, into metabolites.	The transformation of content through personalization, adaptive learning, or symptom-based module delivery.
Excretion	The process by which a drug or its metabolites are eliminated from the body, typically via the kidneys or bile.	The cessation or decay of engagement, including dropout, passive use, or therapeutic saturation.

Unlike molecules, digital therapeutics require belief and trust. The Digital Working Alliance (DWA) formation captures the patient’s perceived alignment with the PDT, including trust, relevance, and shared goals [3]. It modulates each ADME phase, just as bioavailability in pharmacology determines how much of an administered dose enters systemic circulation. High DWA increases absorption by improving the likelihood that patients will start and stick with the PDT. It enhances distribution by encouraging more consistent use, improves metabolism by increasing receptiveness to personalization, and slows excretion by prolonging engagement and benefit. DWA is best treated as a cross-cutting variable in digital PK models, not a separate step, but a scalar that determines whether digital input becomes therapeutic exposure (Table 2).

**Table 2:** Impact of Digital Working Alliance (DWA) on the Digital ADME Framework: An Illustrative Example This author-created table presents a hypothetical example demonstrating how the level of a patient's Digital Working Alliance (DWA) with a prescription digital therapeutic (PDT) can modulate the digital pharmacokinetic (PK) process. Drawing parallels from traditional pharmacology, DWA, representing the patient's perceived trust, relevance, and sense of shared goals with the PDT, influences each phase of the digital absorption, distribution, metabolism, and excretion (ADME) framework. A high DWA facilitates greater initiation and adherence (absorption), promotes consistent engagement across time and contexts (distribution), enhances receptiveness to personalization and adaptation (metabolism), and prolongs engagement by delaying dropout (excretion). Conversely, a low DWA impedes these processes, leading to reduced realized digital exposure despite the prescribed digital dose. This illustrates DWA's critical role as a scalar that determines the extent to which digital input translates into meaningful therapeutic exposure, ultimately impacting clinical outcomes (pharmacodynamics (PD)).

Digital PK/PD	High DWA	Low DWA	DWA Impact on PDT
Patient's State	Trusts content, finds modules relevant, feels shared goals.	Skeptical, finds interface difficult, content feels generic.	DWA modulates how the prescribed digital dose is processed.
Absorption	More likely to complete onboarding and start modules.	Delays or skips onboarding and initial modules.	High DWA enhances initiation and uptake of the digital intervention.
Distribution	Consistently engages with modules at recommended times and contexts.	Inconsistent engagement, uses passively, distracted during sessions.	High DWA promotes consistent and context-appropriate engagement over time.
Metabolism	Receptive to personalized content and adaptations.	Ignores or resists personalized recommendations.	High DWA increases the effectiveness of content transformation and tailoring.
Excretion	Less likely to abandon the program prematurely, sustained engagement.	Prone to early disengagement, dropout, or passive use.	High DWA delays disengagement and reduces attrition or therapeutic decay.
Digital Exposure	Higher realized digital exposure.	Lower realized digital exposure.	High DWA increases the amount of meaningful interaction with the PDT.
Digital PD	Increased likelihood of achieving desired therapeutic outcomes.	Reduced likelihood of achieving desired therapeutic outcomes.	Digital exposure, influenced by DWA, impacts the probability of clinical benefit.

Digital pharmacodynamics: dose-exposure-response curves and closed-loop therapeutics

While PK models delivery and exposure, PD models effect. In drug development, PD describes receptor activity, therapeutic windows, and adverse event profiles [2]. In digital therapeutics, PD must capture how software affects brain function, behavior, symptoms, and outcomes.

PDTs exert effects through multiple pathways, including cognitive restructuring, attentional retraining, biofeedback, relaxation training, and behavioral reinforcement. These actions are dynamic, time-varying, and often personalized. Accordingly, digital response curves must move beyond average treatment effects to individualized trajectories of change.

**Figure 1:**
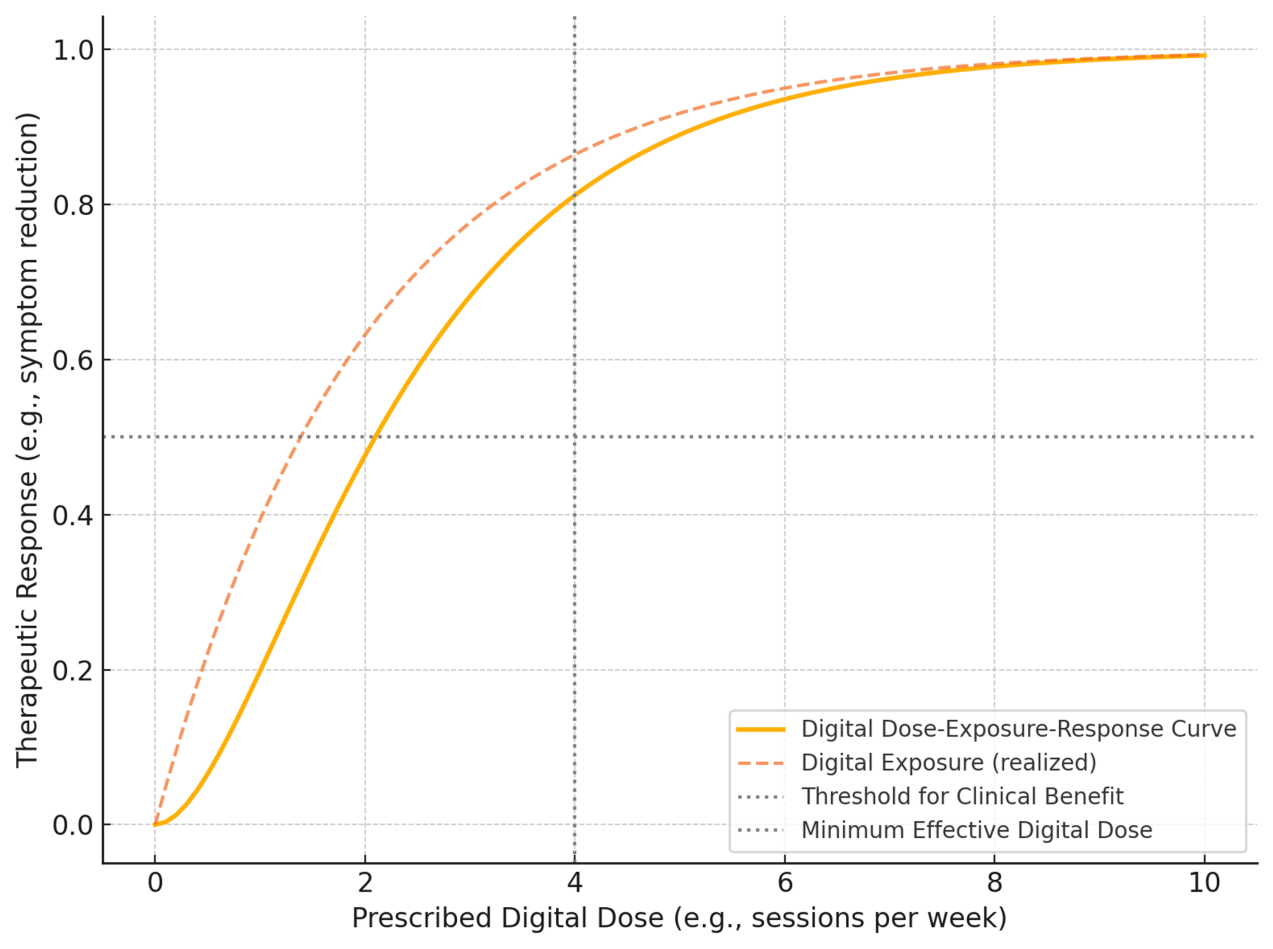
Example Digital Dose-Exposure-Response Curve for a Prescription Digital Therapeutic This conceptual figure illustrates how therapeutic response to a prescription digital therapeutic (PDT) varies as a function of prescribed digital dose and realized digital exposure. The solid curve represents the modeled relationship between increasing prescribed dose (e.g., number of PDT sessions per week) and the resulting clinical response (e.g., symptom reduction). The dashed curve shows realized exposure, which may saturate or diverge from the prescribed dose due to variability in user engagement. The figure highlights key pharmacometric concepts, including the minimum effective digital dose (vertical dotted line) and the threshold for clinical benefit (horizontal dotted line). This digital dose-exposure-response curve enables quantification of therapeutic windows, supports adaptive dosing logic, and provides a foundation for embedding predictive engines within closed-loop digital therapeutic systems.

We define digital PD across several axes. Symptom change involves measurable improvement in clinical endpoints, such as reduced monthly migraine days reduction. Behavioral shift includes altered routines, avoidance patterns, or health behaviors logged by the PDT. Biometric signatures encompass objective signals, such as heart rate variability or sleep cycles, that reflect therapeutic engagement or benefit. Functional outcomes include improvements in quality of life, productivity, and social participation. These outcomes can be plotted against exposure to create digital dose-exposure-response curves (Figure 1). Such curves allow quantification of minimal effective exposure, time to response onset, peak effect timing, and durability of benefit. With sufficient data, PDTs can embed predictive engines that continuously monitor patient input and response, adjusting content in real-time, a hallmark of closed-loop therapeutics.

Closed-loop PDTs behave like biologics with embedded pharmacologic feedback. They sense the patient’s state, deliver a personalized digital dose, monitor the response, and adapt the next intervention. This feedback architecture opens new frontiers for precision psychiatry, neurobehavioral rehabilitation, and self-regulating digital care.

Implications for development, regulation, commercialization, and practice

A formal digital PK/PD model has implications across the lifecycle of PDTs. In clinical development, it enables adaptive trial designs using engagement thresholds as inclusion criteria, supports modeling of responder profiles based on exposure-response curves, and facilitates early identification of digital non-responders for protocol adjustment. In the regulatory strategy, it informs labeling language such as minimum recommended use or expected time to effect, enables post-market comparability analysis of software updates using version-to-version PK equivalence, and justifies adaptive content delivery as titration, akin to pharmacologic adjustments. In commercialization and market access, it supports value-based reimbursement models based on verified engagement and outcome, allows tiering of PDTs within digital formularies based on PK/PD transparency, and enables actuarial forecasting of cost offsets using exposure-linked outcomes. In clinical practice, it equips clinicians with dosing guidance, titration logic, and expected response timelines, supports integration of digital pharmacometrics into electronic health records and care pathways, and enhances clinician confidence by transforming PDTs into active, managed treatments rather than passive tools.

Conclusion

As PDTs mature, they demand the same scientific and regulatory infrastructure that enabled pharmacology to become a disciplined science. By translating concepts like dose, exposure, ADME, and response into digital terms, we enable PDTs to be evaluated, prescribed, and reimbursed with the same rigor as molecular drugs. The digital ADME model captures how therapeutic software flows through the user experience. DWA modulates that flow, shaping whether the prescribed dose becomes meaningful exposure. Digital response curves capture how software changes brain, behavior, and outcomes over time. Together, they form the foundation of digital pharmacometrics. This is not an analogy; it is a necessity. Without PK/PD equivalents, PDTs remain outside the therapeutic mainstream, evaluated as apps rather than agents. With them, we open the door to precision software medicine: measurable, dynamic, trusted, and transformative.
